# How sleep quality affects activities of daily living in Parkinson’s disease: the mediating role of disease severity and the moderating role of cognition

**DOI:** 10.3389/fnagi.2023.1238588

**Published:** 2023-09-29

**Authors:** Yajun Luo, Junyi Liu, Dongqin Chen, Manhua Liu, Yuan Yuan, Jingzhe Hu, Jiayu Wu, Fen Wang, Chunfeng Liu, Juping Chen, Chengjie Mao

**Affiliations:** ^1^Department of Neurology and Clinical Research Center of Neurological Disease, The Second Affiliated Hospital of Soochow University, Suzhou, China; ^2^Department of Neurology, Dushu Lake Hospital Affiliated to Soochow University, Suzhou, China; ^3^Jiangsu Key Laboratory of Neuropsychiatric Diseases and Institute of Neuroscience, Soochow University, Suzhou, China; ^4^Department of Neurology, Changshu Hospital Affiliated to Nanjing University of Chinese Medicine, Changshu, China

**Keywords:** Parkinson’s disease, activities of daily living, disease severity, sleep quality, cognition

## Abstract

**Objective:**

The aim of this study was to explore the influential mechanism of the relationship between sleep quality and activities of daily living (ADL) in patients with Parkinson’s disease (PD), we hypothesized disease severity as a mediator and assumed the mediating process was regulated by cognition.

**Methods:**

194 individuals with PD (95 women and 99 men) were enrolled in study. The Pittsburgh Sleep Quality Index (PSQI) was used to assess sleep quality of PD patients. Patients’ ADL, disease severity and cognition were measured by the Unified Parkinson’s Disease Rating Scale-II (UPDRSII), Hoehn-Yahr (H-Y) Scale, and Mini-Mental State Examination (MMSE). We investigated the mediating role of disease severity and the moderating effect of cognition on the association between sleep quality and ADL in PD patients.

**Results:**

The score of UPDRSII was positively correlated with the score of PSQI and H-Y stage, while the score of MMSE was negatively correlated with the score of H-Y stage and UPDRSII. Sleep quality predicts disease severity, and disease severity predicts ADL. Disease severity mediated the relationship between sleep quality and ADL, and the mediating effect was 0.179. Cognition alone did not affect ADL, but the interaction between disease severity and cognition was significantly affected ADL, confirming the moderating effect of cognition in PD patients.

**Conclusion:**

Disease severity mediated the association between sleep quality and ADL, good cognition significantly reduced disease severity’s mediating influence on the relationship between sleep quality and ADL. Our study indicated a close relationship between ADL and sleep and cognition in PD, and also provided new insights into the overall management of PD and a better quality of life of PD patients.

## 1. Introduction

Parkinson’s disease (PD) is the second largest neurodegenerative disease after Alzheimer’s disease at present. It is estimated will affect about 17 million people in the world by 2040 (Dorsey et al., 2018). In addition to the typical motor symptoms such as static tremor, rigidity, and bradykinesia, non-motor symptoms (NMS) such as sleep disorders, cognitive decline, anxiety, and depression are also commonly seen and have a negative impact on patients’ quality of life in PD.

Activities of daily living (ADL) refers to the necessary activities that a person performs every day to meet the needs of daily life. It includes basic skills such as dressing, bathing, eating, and other complex activities like social communication, which is closely related to the quality of life. In the PD population, disease severity seriously affects ADL. With the progress of PD, ADL ability decreases (Hariz and Forsgren, 2011; Bouça-Machado et al., 2022).

Studies have also shown that PD patients with poorer sleep quality and cognition usually have a lower ability to life (Thorpe et al., 2016; Çavuşoğlu et al., 2021). PD itself may interact with sleep, and patients with sleep disorders always have the more progressive aggravation of neurodegenerative processes (Ju et al., 2014; Schütz et al., 2022) which seriously reduces the patient’s quality of life (Thorpe et al., 2016). Sleep alterations also occur before the motor alterations in PD. Rapid eye movement (REM) behavior disorder (RBD) is a potential prodromal biomarker to PD, it is reported RBD can cause alpha-synuclein deposition and neuroinflammation, and accelerating the progression of PD, besides, circadian rhythm injury can also contribute to abnormal inflammatory reaction under the pathogenesis of PD, glymphatic system dysfunction plays a pathogenic role in it (Scott-Massey et al., 2022). Sleep disorder greatly increased risk of PD.

Cognitive impairment can be seen at an early stage in PD. Any types of cognitive impairment are closely related to the severity of PD measured by the Hoehn-Yahr (H-Y) stage and can lead to the deterioration of neuropsychology and quality of life (Simon-Gozalbo et al., 2020). It was proposed that cognitive deficits can affect the awareness of ADL, resulting in the impairment of ADL capability (Hartle et al., 2022).

In view of the effect of disease severity, sleep quality, and cognitive function on ADL and clinical manifestations in PD patients, we have no doubt that in PD patients, disease severity, sleep quality, and cognitive function all have an impact on ADL. But how disease severity, sleep quality, and cognitive function overall affect ADL is still unclear.

After searching the relevant literature, we did not find any research about the synthetical influence of sleep quality, disease severity, and cognition on the ADL of PD. However, a better understanding of how they interact is very important and will help us to improve the ADL of patients, especially the early PD patients, and eventually to improve the quality of life in PD patients.

**Figure 1:**
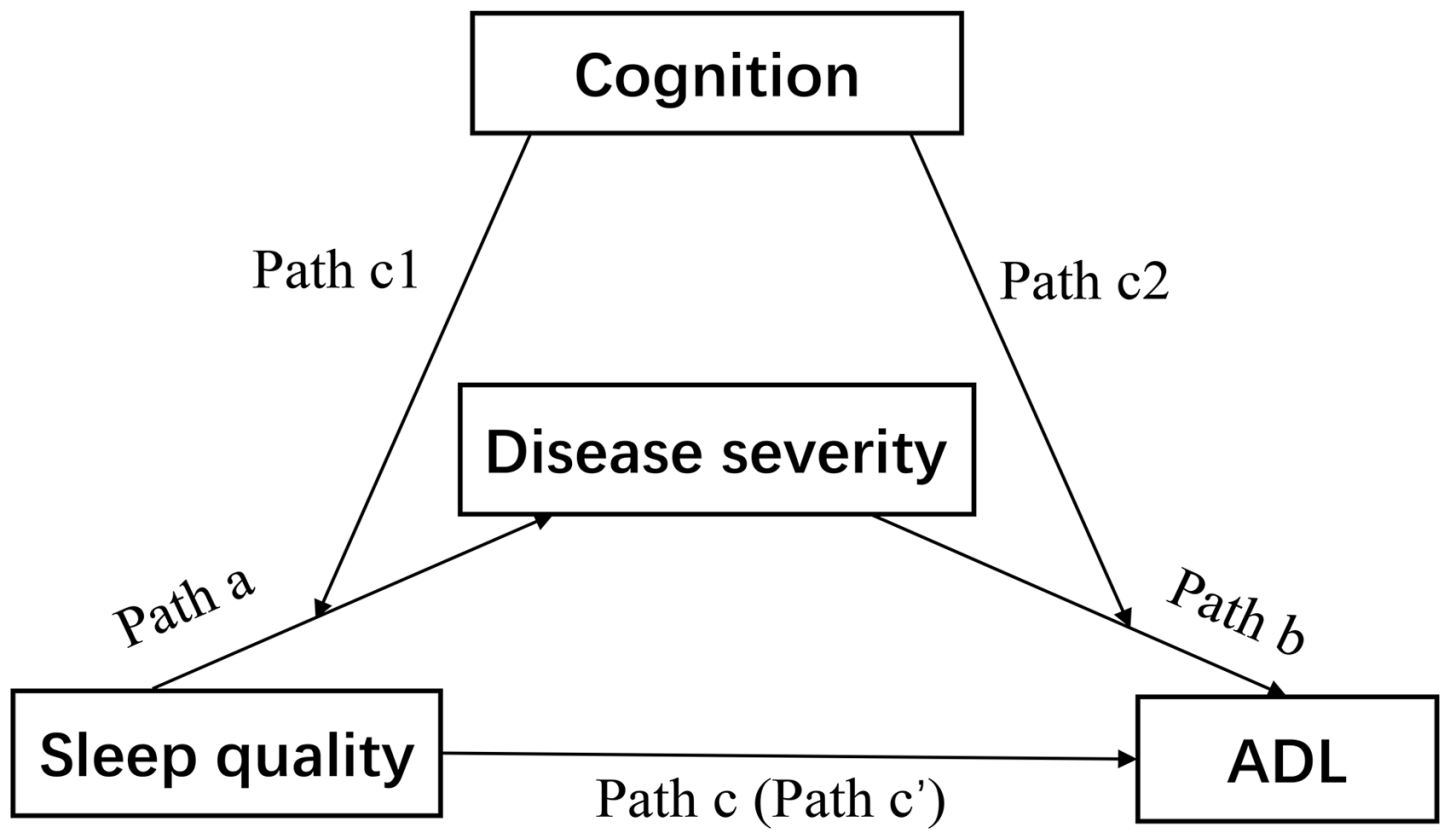
Theoretical model of the study. The association between sleep quality and ADL was the total effect (Path c), which consisted of a direct effect of sleep quality (Path c’) and an indirect effect via disease severity (Path a × Path b). ADL, activities of daily living.

Therefore, the aim of this study was to explore the interrelationships between sleep quality, disease severity, cognition, and ADL in PD patients. We hypothesized that in the population of PD, sleep quality and disease severity are associated with ADL, and assumed the mediating process is regulated by cognition (Figure 1).

## 2. Materials and methods

### 2.1. Participants

A total of 207 patients in the Parkinson’s specialist outpatient departments of Second Affiliated Hospital of Soochow University were enrolled in our study from August 2017 to July 2022. All patients met the inclusion and exclusion criteria, among them, 13 were excluded due to incomplete data, 194 patients ultimately included in the study. Participants were eligible for the study if they met the following conditions: (a) were diagnosed based on the Movement Disorder Society (MDS) Clinical Diagnostic Criteria for PD (Postuma et al., 2015), (b) regular treatment ≥3 months, and (c) able to understand and complete all scales. Exclusion criteria were as follows: (a) were diagnosed with Parkinsonism-plus syndrome, such as multiple system atrophy, progressive supranuclear palsy, (b) secondary Parkinsonism induced by organic disorders or traumatic brain injury, (c) had a history of deep brain stimulation surgery, (d) presence of other diseases involving ADL, for example, disability, and (e) were suffering from severe visceral diseases including cardiovascular, cerebrovascular, kidney diseases. This study was approved by the ethics committee of the Second Affiliated Hospital of Soochow University (JD-LK-2021-132-02) and informed consent was obtained from all participants. All scales were completed by a neurologist with professional knowledge of movement disorders, and data was collected through face-to-face conversation with patients.

### 2.2. Patient assessment

Disease severity was evaluated according to the Hoehn and Yahr (H-Y) scale (Hoehn and Yahr, 1967), the H-Y scale has remained the most commonly used scale to describe the severity of PD worldwide (Goetz et al., 2004), higher scores indicated severe disease severity.

The Pittsburgh Sleep Quality Index (PSQI) was used to assess sleep quality in patients with PD in a recent month. It consists of 19 items and generates 7 dimensions as follows: subjective sleep quality, sleep duration, medication use, sleep latency, efficiency of sleep, sleep disturbances, daytime dysfunction, and each dimension takes score from zero to three (Hashemzadeh et al., 2022). PSQI was reliable in distinguishing good and bad sleep habits, and showed strong reliability and validity in a variety of samples (Mollayeva et al., 2016).

The Mini-Mental State Examination (MMSE) (Folstein et al., 1983) was used to assess cognitive function, which was composed of five testing categories including orientation, memory registration, memory recall, calculation, attention, and language (Wei et al., 2022). MMSE was widely used in the cognitive evaluation of PD, and higher scores indicated better cognition.

ADL was accessed according to The Unified Parkinson’s Disease Rating Scale (UPDRS II) (Movement Disorder Society Task Force on Rating Scales for Parkinson’s Disease, 2003), which was specifically designed for the PD population and had sufficient internal consistency and convergent validity, lower UPDRS II score represented less disability in performing ADL in PD (Bouça-Machado et al., 2022).

All patients were at “on” stage, and the study was approved by the Ethics Committee of the Second Affiliated Hospital of Soochow University.

### 2.3. Data analysis

All statistical analyses were performed using SPSS version 26.0, we compared the demographic and general characteristics of patients and was compared using independent samples *t*-test or Mann–Whitney *U* test for continuous variables (depending on whether the normality assumption was tenable), and Chi-square test was used for categorical variables. The zero-order correlations between variables were calculated. The models were developed with PSQI as the independent variables, UPDRS II as the dependent variables, and MMSE as a mediator, and then were tested using Hayes’ PROCESS macro 3.5 for SPSS (model 4 and model 58). According to previous studies, there are significant gender differences in ADL and sleep quality in PD patients, so we added gender as a control variable to reduce confounding bias (Lubomski et al., 2014; Mahale et al., 2021). The following path was analyzed: (a) the mediating role of disease severity in the relationship between sleep quality and ADL; and (b) the moderated mediation of MMSE between disease severity and ADL.

The mediation and moderation effects were tested using bias-corrected bootstrapping (*n* = 5,000) and 95% confidence intervals (CI), if a 95% bootstrapped CI does not include zero, it means the parameter is statistically significant. A *p-*values less than 0.05 were considered statistically significant.

## 3. Results

### 3.1. Demographic and general characteristics of PD patients

194 patients with PD participated in this study, women accounted for 49% of the total sample and men accounted for 51%. The mean age of all patients was 65.40 ± 9.44 years old, and 86.6% of the patients had received primary education or above. The median H-Y stage of all patients was 2 (1.5–3.0). Table 1 shows the demographic and general characteristics of the participants.

**Table 1:** The demographic and general characteristics of the participants (*N* = 194).

Variable	Mean/Median	N (%)	Min	Max
Sex				
Male		99 (51.00)		
Female		95 (49.00)		
Age (years)	65.40 ± 9.44		34	87
34–50		9 (4.64)		
51–70		126 (64.95)		
71–87		59 (30.41)		
Education (years)	8 (4–12)		0	18
Uneducated		26 (13.40)		
Primary school		58 (29.89)		
Middle school		56 (28.87)		
High school and above		54 (27.84)		
LEDD (mg)	400 (300–600)		0	1696
0–500		132 (68.04)		
501–1000		49 (25.26)		
1001–1500		10 (5.15)		
1501–1696		3 (1.55)		
Disease duration (months)	14 (10–24)		1	158
1–60		170 (87.63)		
61–120		21 (10.82)		
121–158		3 (1.55)		
MMSE	24.43 ± 5.06		3	30
PSQI	9.62 ± 3.82		1	25
H-Y	2 (1.5–3.0)		1	5
UPDRSII	11.06 ± 6.95		0	35

Continuous variables are expressed as mean ± standard deviation or as median (interquartile range). MMSE, Mini-Mental State Examination; PSQI, The Pittsburgh Sleep Quality Index; HY, Modified Hoehn and Yahr Scale; UPDRS II, Unified Parkinson’s Disease Rating Scale.

Besides, according to the study results on validity and reliability of Chinese version (Mao et al., 2018), we categorized patients into two groups and compared them. Scores ≤7 indicating good sleepers, and > 7 indicating poor sleepers (Table 2). Comparing with patients with good sleep, patients with poor sleep have a greater UPDRS II score, an older age and a shorter disease duration. There were no significant differences in sex, education, levodopa equivalent daily dosage (LEDD), MMSE, H-Y stage between the two groups.

**Table 2:** The demographic and general characteristics in good and poor sleepers.

Variable	Patients with poor sleep (*N* = 138)	Patients with good sleep (*N* = 56)	*p* value
Male sex	71 (51.4)	28 (50.0)	0.86
Age (years)	67.3 ± 9.2	60.8 ± 8.5	<0.001
Education (years)	8 (3.0, 12.0)	9 (6.0, 9.8)	0.39
LEDD (mg)	400 (300.0, 600.0)	362.5 (253.1, 562.5)	0.36
Disease duration (months)	14 (10.0, 17.3)	24 (11.3, 68.8)	<0.001
MMSE	25 (22.0, 28.0)	27 (24.0, 28.0)	0.06
H-Y	2.25 (2.0, 3.0)	2 (1.5, 2.9)	0.06
UPDRSII	10 (8.0, 15.0)	8 (3.3, 11.0)	<0.001

Demographic and general characteristics were compared using independent samples *t*-test or Mann–Whitney *U* test for continuous variables (depending on whether the normality assumption was tenable), and Chi-square test was used for categorical variables. MMSE, Mini-Mental State Examination; PSQI, The Pittsburgh Sleep Quality Index; HY, Modified Hoehn and Yahr Scale; UPDRS II, Unified Parkinson’s Disease Rating Scale.

### 3.2. Descriptive statistics and bivariate correlations between sleep quality, motor symptoms, cognition, and ADL in PD patients

Pearson correlation analysis was performed on sleep quality, disease severity, ADL, and cognition. Table 3 shows the bivariate correlations between study variables, and includes their respective means and standard deviations. The table shows significant positive associations between UPDRSII and both PSQI and H-Y stage, and shows significant negative associations between MMSE and both H-Y stage and UPDRSII. No correlation is found between MMSE and PSQI.

**Table 3:** Means, standard deviations, and correlations between study variables.

Variable	PSQI	H-Y	UPDRSII	*M*	SD
PSQI (Sleep quality)	–	0.166*	0.270***	9.62	3.82
H-Y (Disease severity)	0.166*	–	0.614***	2.22	0.77
UPDRSII (ADL)	0.270***	0.614***	–	11.06	6.95
MMSE (Cognition)	−0.137	−0.263***	−0.245***	24.43	5.06

***p < 0.001, *p < 0.05, two-tailed. MMSE, Mini-Mental State Examination; PSQI, The Pittsburgh Sleep Quality Index; HY, Modified Hoehn and Yahr Scale; UPDRS II, Unified Parkinson’s Disease Rating Scale; ADL, activities of daily living.

### 3.3. Moderated mediation model analysis

A moderated mediation model was calculated to test whether cognition would moderate the indirect relationships between sleep quality and ADL via disease severity.

#### 3.3.1. PD severity has a mediating role between sleep quality and ADL

**Figure 2:**
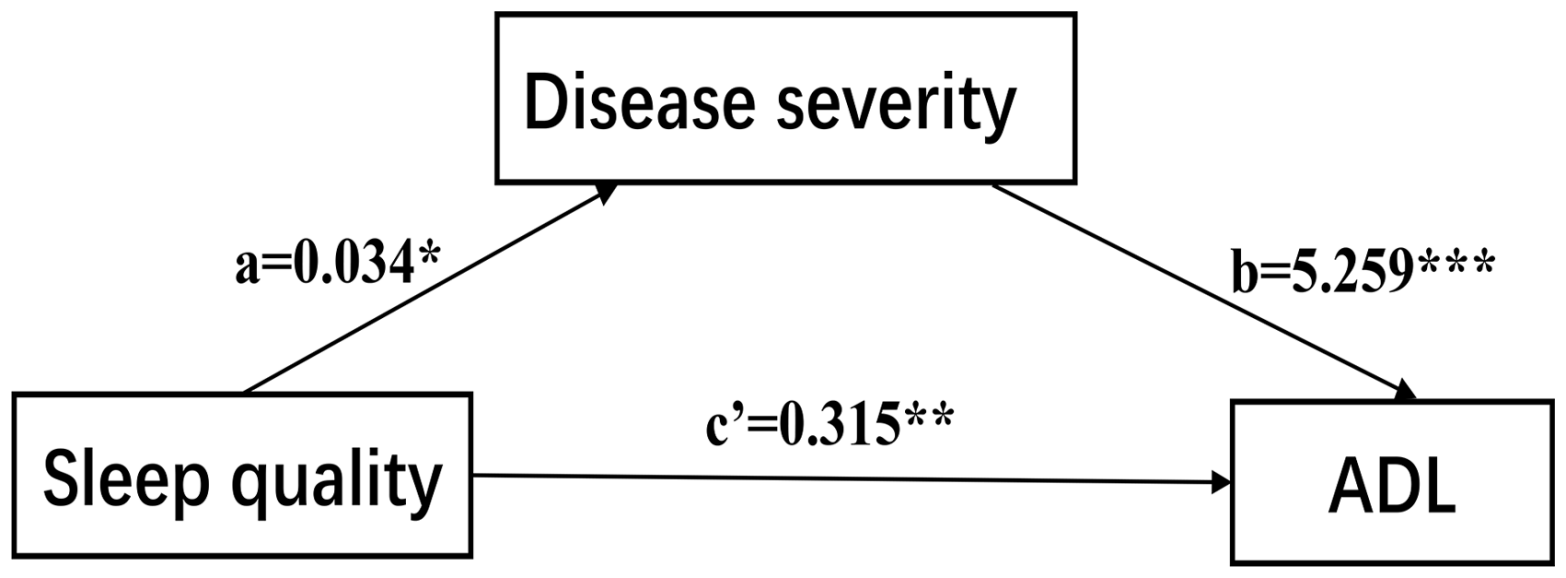
The confirmed mediation model. ADL, activities of daily living. ***p < 0.001; **p < 0.01; *p < 0.05.

We used Model 4 of Hayes’s Process first to investigate the mediation effect of disease severity (Figure 2), the results indicated that PSQI had a significant positive effect on UPDRS II (Path c: *B* = 0.496, SE = 0.126, *p* < 0.001, 95% CI [0.247, 0.744]), when the H-Y stage was included as a mediator, the results showed that PSQI still had a significant positive effect on UPDRS II (Path c’: *B* = 0.315, SE = 0.103, *p* < 0.01, 95% CI [0.111, 0.518]), PSQI positively predicts HY stage (Path a: *B* = 0.034, SE = 0.014, *p* < 0.05, 95% CI [0.006, 0.063]), and H-Y stage positively predicts UPDRS II (Path b: *B* = 5.259, SE = 0.516, *p* < 0.001, 95% CI [4.242, 6.277]), the indirect effect is 0.034*5.259 = 0.179, 95%CI [0.039, 0.358], which did not include 0. Therefore, PD severity has a mediating role between sleep quality and ADL, and the mediating effect accounted for 36% of the total effects.

#### 3.3.2. Association between disease severity and ADL was moderated by cognition

**Figure 3:**
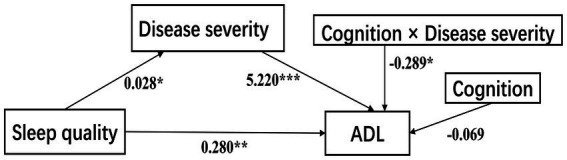
The confirmed moderated-mediation model. ADL, activities of daily living. ***p < 0.001; **p < 0.01; *p < 0.05.

Model 58 of Hayes’s Process was applied to test our theoretical moderated-mediation model. As Table 4 shows, the direct relationship of sleep quality to ADL was significant (*B* = 0.280, SE = 0.103, *p* < 0.01, 95% CI [0.078, 0.483]), disease severity had statistically significant effects on ADL (*B* = 5.220, SE = 0.529, *p* < 0.001, 95% CI [4.176, 6.264]), cognition alone did not affect ADL (*B* = −0.069, SE = 0.080, 95% CI [−0.228, 0.089]), but the interaction between disease severity and cognition was significantly affected ADL (Path c2: *B* = −0.289, SE = 0.121, *p* = 0.018, 95% CI [−0.528, −0.050]), which indicates that the association between disease severity and ADL was moderated by cognition. However, another interaction term showed that sleep quality did not interact with cognition to predict disease severity (path c1: *B* = −0.003, SE = 0.003, 95% CI [−0.009, 0.003]), thus not supporting the moderation effect in this path. Sex was significant when disease severity was a dependent variable, but was insignificant when ADL was analyzed as a dependent variable (Figure 3).

**Table 4:** Summary of the moderated-mediation model.

Antecedent		Consequent									
		HY					UPDRSII				
		B	SE	*p*	LLCI	ULCI	B	SE	*p*	LLCI	ULCI
HY	b	–	–	–	–	–	5.220	0.529	0.000	4.176	6.264
HY*MMSE	c2	–	–	–	–	–	−0.289	0.121	0.018	−0.528	−0.050
PSQI*MMSE	c1	−0.003	0.003	0.296	−0.009	0.003	–	–	–	–	–
PSQI	a/c’	0.028	0.014	0.047	0.0003	0.055	0.280	0.103	0.007	0.078	0.483
MMSE		−0.038	0.010	0.0004	−0.059	−0.017	−0.069	0.080	0.390	−0.228	0.089
Sex		−0.236	0.106	0.027	−0.444	−0.028	−0.117	0.777	0.880	−1.649	1.415
Constant		0.343	0.166	0.040	0.015	0.672	10.942	1.230	0.000	8.516	13.368
		*R*^2^ = 0.116					*R*^2^ = 0.429				
		*F* = 6.18, *p* < 0.001					*F* = 28.22, *p* < 0.001				

MMSE, Mini-Mental State Examination; PSQI, The Pittsburgh Sleep Quality Index; HY, Modified Hoehn and Yahr Scale; UPDRS II, Unified Parkinson’s Disease Rating Scale; B, Standard coefficients; SE, Standard Error; p, *p* value; LLCI, Lower level 5% confidence interval; ULCI, Upper level 95% confidence interval.

#### 3.3.3. Correlation between UPDRS II and H-Y stage was stronger in people with low cognition

We calculated effects at different levels of cognition (1 SD below the mean, mean, 1 SD above the mean), and the mediating effect value of disease severity between sleep quality and ADL is as follows. Under a 95% bootstrapped CI, the indirect effect reached statistical significance for the mean (95% CI [4.176, 6.264]), high (+1 SD) (95% CI [2.248, 5.267]) and low (−1 SD) (95% CI [5.001, 8.362]) cognition values (Table 5). When cognition is perceived to be low (−1 SD), severer H-Y stage is positively associated with poorer ADL (Effect = 6.682, *p* < 0.001), indicating that the direct relationship between disease severity and ADL has become weaker with an increase in cognition. Thus, cognition is a protective component that can effectively reduce the harmful consequences of poor sleep quality on ADL.

**Table 5:** Conditional effects of HY on UPDRSII at different values of MMSE.

MMSE	Effect	Boot SE	*p*	LLCI	ULCI
Low (−1SD)	6.682	0.105	0.000	5.001	8.362
Medium	5.220	0.080	0.000	4.176	6.264
High (+1SD)	3.758	0.063	0.000	2.248	5.267

LLCI, Lower level 5% confidence interval; ULCI, Upper level 95% confidence interval; MMSE, Mini-Mental State Examination; HY, Modified Hoehn and Yahr Scale; UPDRS II, Unified Parkinson’s Disease Rating Scale.

## 4. Discussion

In this study, we tested a model of interactions between sleep quality, disease severity, and ADL in PD patients. This study revealed that cognition played a role of moderator which confirmed our hypothesis. Sleep quality directly affected ADL and indirectly affected ADL via disease severity, furthermore, cognition moderated the association between sleep quality and ADL, and the indirect effect gradually decreased as a score of MMSE increased, indicating that better cognition played the role in moderating and palliating between sleep quality and ADL. In other words, patients with poor sleep quality affect ADL by aggravating the severity of the disease, and better cognition plays a role in buffering these negative effects.

The evaluation of sleep disorders is increasingly recognized as an important aspect of the health of patients with chronic diseases. Firstly, consistent with recent studies, our results indicated that poor sleep quality could be a risk factor for decreased ADL ability in PD patients (Liguori et al., 2021; Yi et al., 2022). Sleep quality can affect ADL in multiple ways, Arias-Fernández et al. (2021) found poor sleep quality was associated with impaired physical function and were linked to muscle weakness in older adults. Rolinski et al. (2014) found PD patients with RBD related to a higher prevalence of mood disorders and a greater impact on ADL. Poor sleepers usually displayed greater anxiety and also fatigue, serotonin is thought to be a major factor in this occurrence. Besides, in an early PD cohort, clear association had been found between poor sleep quality and worsening depression over time, so it still needs to be explored further to determine if these symptoms play important roles in the path sleep quality affect ADL (Koh et al., 2022). PD patients with sleep disorders often experienced more NMS than those without sleep disorders (Neikrug et al., 2014), it has been demonstrated that poor sleep can also aggravate pain and cognitive impairment, while these NMS are important determinant factors of the quality of life in PD patients (Rana et al., 2018; Santos García et al., 2021). So, we considered sleep quality as an important factor which affects ADL. In addition to motor symptoms, the overall consideration of the impact of non-motor symptoms, including sleep disturbance and cognition, on quality of life is worthy of future research.

Secondly, we found poor sleep quality was associated with more severe PD stages. The connection between sleep and neurodegenerative disorders has been highlighted in many studies (Valdinocci et al., 2017; Scott-Massey et al., 2022; Kopeć et al., 2023; Wang et al., 2023). Mechanisms behind sleep quality influencing disease severity in neurodegenerative diseases remain speculative: Accumulation of neurotoxic proteins, including α-synuclein (α-syn) in PD and amyloid β-protein (Aβ) and tau in Alzheimer’s disease (AD), is a common pathological mechanism. In AD, poor sleep may increase neuronal activity, and decrease the clearance efficiency of extracellular metabolites, which may contribute to Aβ and tau accumulation (Minakawa, 2022). As for α-syn, research in mice and humans proved that persistent wakefulness due to impaired sleep can raise the extracellular soluble α-syn in the interstitial fluid (ISF) and cerebrospinal fluid (CSF) (Holth et al., 2019) and accelerates PD progression. Glymphatic system dysfunction plays a pathogenic role in it. The main function of the glymphatic system is to facilitate the removal of pathological proteins and metabolites to optimize neurological functions, it was reported bidirectional relationships exist between α-syn, glymphatic system and sleep (Scott-Massey et al., 2022). Sleep disruption impairs the glymphatic system and may lead to future abnormal α-syn aggregation, and α-syn accumulation further causes the suppressed glymphatic clearance, then, the impaired glymphatic clearance further aggravates α-syn deposition and PD pathology (Zhang et al., 2023). Moreover, the interplay between sleep and neuroinflammatory reaction and nigral-dopaminergic neuronal system degeneration may also aggravate motor impairment (Lauretti et al., 2017; Zou et al., 2019; Ding et al., 2021), thus affect disease severity in PD. In our model, we pointed out that disease severity plays an indispensable part in mediating the association between sleep quality and ADL, but it only mediates this relationship to a limited extent. This suggests clinical practice or future research should explore additional mediators in order to offer a more comprehensive view of the complex link between sleep quality and ADL.

Finally, our study showed that cognition played a moderating role in the relationship between ADL and PD severity. Cognition deficit is one of the major clinical non-motor symptoms of PD, about 40% of PD patients at an earlier stage have mild cognitive impairment (MCI), and approximately 40% of PD-MCI progress to Parkinson’s disease dementia (PDD) over 3 years (Aarsland and Kurz, 2010; Weintraub et al., 2018). Cognitive deficits were proved to be related to ADL difficulties in PD population (Rosenthal et al., 2010). Basic ADL, instrumental activities, and advanced activities are three items stratified by ADL. Previous studies showed the appearance of cognitive dysfunction usually impairs advanced activities at first and then affects basic activities with the decline of cognitive ability (De Vriendt et al., 2012). Besides, cognition deficit is also associated with severity of PD, patients with PD-MCI and PDD always present a higher score of H-Y scale, as well as a worse motor and functional levels, which aggravates the decline of ADL capability (Happe et al., 2002; Lysen et al., 2019; Simon-Gozalbo et al., 2020; Yi et al., 2022). These studies suggested that early prevention and intervention of cognitive deficits are necessary to keep ADL capability.

A recent study showed that cognition decline can mediate the impact of sleep quality on the H-Y stage in PD patients, and was considered to be caused by impairment of the nigrostriatal dopaminergic system (Pfeiffer, 2016; Yi et al., 2022). However, in our study, we put cognition as a moderator, not a mediator, and did not find its moderating effect between sleep quality and the H-Y stage, which may indicate that cognition plays a role of a mediator not a moderator in the relationship between sleep quality and the H-Y stage.

Our findings have theoretical and practical implications for improving ADL in PD patients. In theory, the model explains the correlation mechanism behind sleep quality, disease severity, ADL, and cognition, and lays a foundation for the next step of research. In practice, the results are instructive for improving ADL in PD, intervention for sleep and cognition decline may be an appropriate choice to alleviate ADL decline.

There are several limitations to our study. First, data analysis showed that the mean MMSE score was 24.43 ± 5.06, median H-Y stage was 2 (1.5–3.0), which indicates that most patients have the good cognitive ability and are in the early stage of PD disease. It is difficult to enroll patients of H-Y stage 4–5 due to their limited cognitive and behavioral abilities, so our conclusion may more appropriate for PD patients in an early stage. Second, PSQI has relatively low sensitivity and specificity as a self-report questionnaire, it is necessary to evaluate the sleep quality of patients from the perspective of caregivers. Sometimes the patient said he had a good night, but the caregivers clearly know he had a bad night, which is common in the outpatient clinic. Further studies using more objective methods, such as polysomnography, are needed to investigate the relationship between sleep and ADL. Third, data were cross-sectional and acquired from only one center, the cross-sectional data made making causal inferences between the identified variables and ADL challenging, further longitudinal studies and sample size expansion need to be performed to investigate the causal link between sleep quality and ADL. Finally, we did not have information on variables including depression, anxiety, apathy, all of which could affect ADL, so there may be residual confounding factors that we have not controlled for, additional research is required to identify other potential mechanisms, so as to improve our model.

## 5. Conclusion

Despite the limitations above, our study tested and expanded an intervention perspective model of sleep quality, ADL, disease severity and cognition, indicated a close relationship between ADL and sleep and cognition in PD, and also provided new insights into the overall management of PD and a better quality of life of PD patients.

## Data availability statement

The original contributions presented in the study are included in the article, further inquiries can be directed to the corresponding authors.

## Ethics statement

This study was approved by the ethics committee of the Second Affiliated Hospital of Soochow University (JD-LK-2021-132-02) and informed consent was obtained from all participants.

## Author contributions

YL and CM conceived and designed the study. YL, JL, and DC collected the data, analyzed the data, and drafted the manuscript. ML, YY, JH, JW, and FW collected the data. CM, CL, and JC critically revised it. All authors contributed to the interpretations of the findings and reviewed the manuscript.
